# Proteome analysis reveals roles of L-DOPA in response to oxidative stress in neurons

**DOI:** 10.1186/1471-2202-15-93

**Published:** 2014-07-31

**Authors:** Mohammad-Saeid Jami, Ramavati Pal, Esthelle Hoedt, Thomas A Neubert, Jan Petter Larsen, Simon Geir Møller

**Affiliations:** Department of Biological Sciences, St John’s University, New York, NY USA; Kimmel Center for Biology and Medicine at the Skirball Institute and Department of Biochemistry and Molecular Pharmacology, New York University School of Medicine, New York, NY USA; The Norwegian Centre for Movement Disorders, Stavanger University Hospital, Stavanger, Norway; Cellular and Molecular Research Center, School of Medicine, Shahrekord University of Medical Sciences, Shahrekord, Iran

**Keywords:** Parkinson’s disease, Oxidative stress, Dopamine, Proteomics

## Abstract

**Background:**

Parkinson’s disease (PD) is the second most common neurodegenerative movement disorder, caused by preferential dopaminergic neuronal cell death in the substantia nigra, a process also influenced by oxidative stress. L-3,4-dihydroxyphenylalanine (L-DOPA) represents the main treatment route for motor symptoms associated with PD however, its exact mode of action remains unclear. A spectrum of conflicting data suggests that L-DOPA may damage dopaminergic neurons due to oxidative stress whilst other data suggest that L-DOPA itself may induce low levels of oxidative stress, which in turn stimulates endogenous antioxidant mechanisms and neuroprotection.

**Results:**

In this study we performed a two-dimensional gel electrophoresis (2DE)-based proteomic study to gain further insight into the mechanism by which L-DOPA can influence the toxic effects of H_2_O_2_ in neuronal cells. We observed that oxidative stress affects metabolic pathways as well as cytoskeletal integrity and that neuronal cells respond to oxidative conditions by enhancing numerous survival pathways. Our study underlines the complex nature of L-DOPA in PD and sheds light on the interplay between oxidative stress and L-DOPA.

**Conclusions:**

Oxidative stress changes neuronal metabolic routes and affects cytoskeletal integrity. Further, L-DOPA appears to reverse some H_2_O_2_-mediated effects evident at both the proteome and cellular level.

## Background

Parkinson’s disease (PD) is the second most common neurodegenerative movement disorder, affecting approximately 1% of individuals older than 60 years [1]. The occurrence of PD will most likely double within the next two decades due to an increase in the aging population [2]. PD is caused by preferential dopaminergic neuronal cell death in the substantia nigra, resulting in a reduced level of dopamine in the striatum. By the time of clinical diagnosis this region of the brain has irreversibly lost 50–70% of its neurons compared to unaffected individuals [3]. Despite PD being a complex and multifactorial disease, oxidative stress and mitochondrial dysfunction are thought to be major causes of neurodegeneration in PD [4]. In both idiopathic and genetic PD, oxidative stress is thought to be a common denominator and the substantia nigra of PD subjects exhibit increased levels of oxidized lipids [5], proteins and DNA [6] and decreased levels of reduced glutathione (GSH) [7].

L-3,4-dihydroxyphenylalanine (L-DOPA) remains the most common PD medication as it is converted into dopamine in dopaminergic neurons temporarily relieving motor symptoms. Despite its widespread use there is clear controversy in the field. Pre-clinical *in vivo* studies have shown that L-DOPA can damage dopaminergic neurons due to oxidative stress and perhaps through other mechanisms [8, 9]. Studies have shown that direct intraniagral infusion of L-DOPA in rats results in reduced dopaminergic neuron numbers [10] whilst other studies have demonstrated that L-DOPA increases the levels of nitric oxide in the substantia nigra and striatum [11, 12]. In contrast, many *in vitro* studies have shown that L-DOPA may be neuroprotective through decreased lipid peroxidation [13, 14]. Furthermore, studies have indicated that L-DOPA can act as a neuroprotective agent reducing toxicity evoked by stronger oxidants [15]. Indeed, whether L-DOPA is neurotoxic, or has little to no effect on dopaminergic neuron survival, still remains unanswered [16].

Despite numerous studies, the molecular interplay between oxidative stress and L-DOPA remains fragmented. In this study we have performed a two-dimensional gel electrophoresis (2DE)-based proteomic study to gain further insight into the effects of L-DOPA in response to H_2_O_2_-mediated oxidative stress in SH-SY5Y neuronal cells. We show that L-DOPA influences proteome changes in response to oxidative stress leading to a lowering of reactive oxygen species (ROS) and increased cell survival indicative of a role in neuronal cell protection.

## Results and discussion

### Cell morphology, viability and proteomic profiling of neuronal cells in response to H_2_O_2_ and L-DOPA treatment

**Figure 1:**
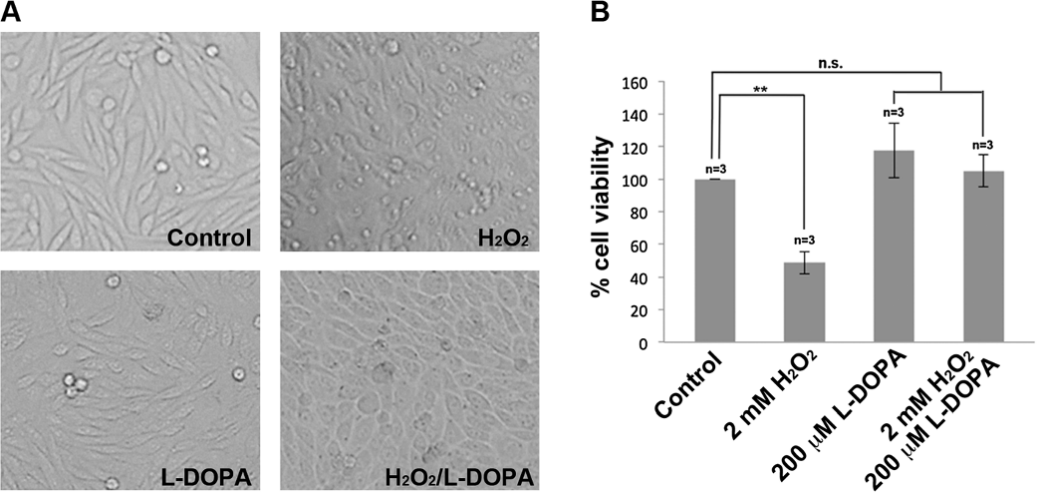
**Cell morphology and cell viability of SH-SY5Y cells in response to H**_**2**_ **O**_**2**_ **, L-DOPA and H**_**2**_ **O**_**2**_ **/L-DOPA treatments. (A)** Cell morphology of SH-SY5Y cells in response to eight hours of 2 mM H_2_O_2_, 200 μM L-DOPA and 2 mM H_2_O_2_/200 μM L-DOPA treatments. **(B)** Neutral red cell viability assays of SH-SY5Y cells in response to eight hours of 2 mM H_2_O_2_, 200 μM L-DOPA and 2 mM H_2_O_2_/200 μM L-DOPA treatments. Each data point is the average of three replicate samples and presented as means ± SD. Standard deviations are indicated by error bars. **p < 0.01.

In order to provide a global overview regarding the mechanism in which L-DOPA may influence toxic effects of H_2_O_2_ in neuronal cells we performed cell viability analysis and subsequent proteomic analyses using SH-SY5Y cells. Although primary neurons or dopaminergic neurons would be ideal for this study in relation to PD we selected SH-SY5Y cells based on reproducibility and because they are dopamine beta hydroxylase active. SH-SY5Y cells were grown under control conditions and were exposed to 2 mM H_2_O_2_, 200 μM L-DOPA or a combination of the two treatments (2 mM H_2_O_2_/200 μM L-DOPA) for eight hours. Cell morphology analysis (Figure 1A) and cell viability assays (Figure 1B) demonstrated that SH-SY5Y cells exposed to L-DOPA showed no effect on morphology or cell viability (Figure 1). By contrast, H_2_O_2_ exposure decreased cell viability, but this effect was reversed in response to co-treatment with L-DOPA (Figure 1). This suggested that L-DOPA might have a protective effect towards excess oxidative stress.

**Figure 2:**
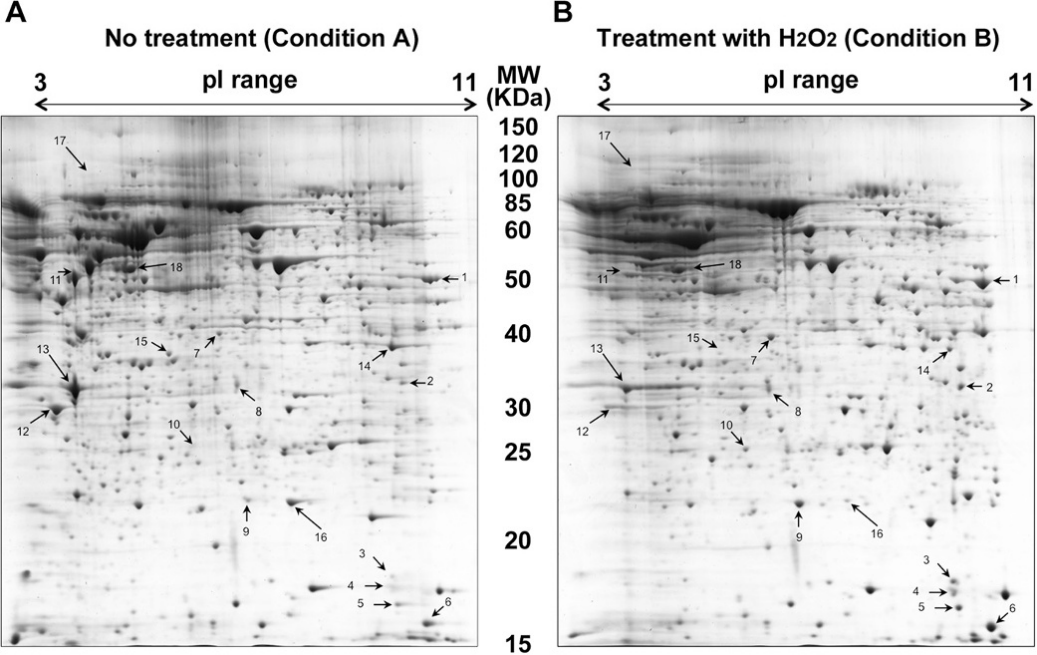
**Comparison of the proteomes of SH-SY5Y cells with or without H**_**2**_ **O**_**2**_ **.** Representative 2-DE gels (n = 3 for each treatment) of the proteomes of SH-SY5Y cells grown for 8 hours in the **(A)** absence or **(B)** presence of 2 mM H_2_O_2_. The uppercase letters are used for those spots overrepresented in each condition whereas lowercase letters are used for spots underrepresented. The spots differentially represented are numbered and correspond to the proteins listed in Table 2.

**Figure 3:**
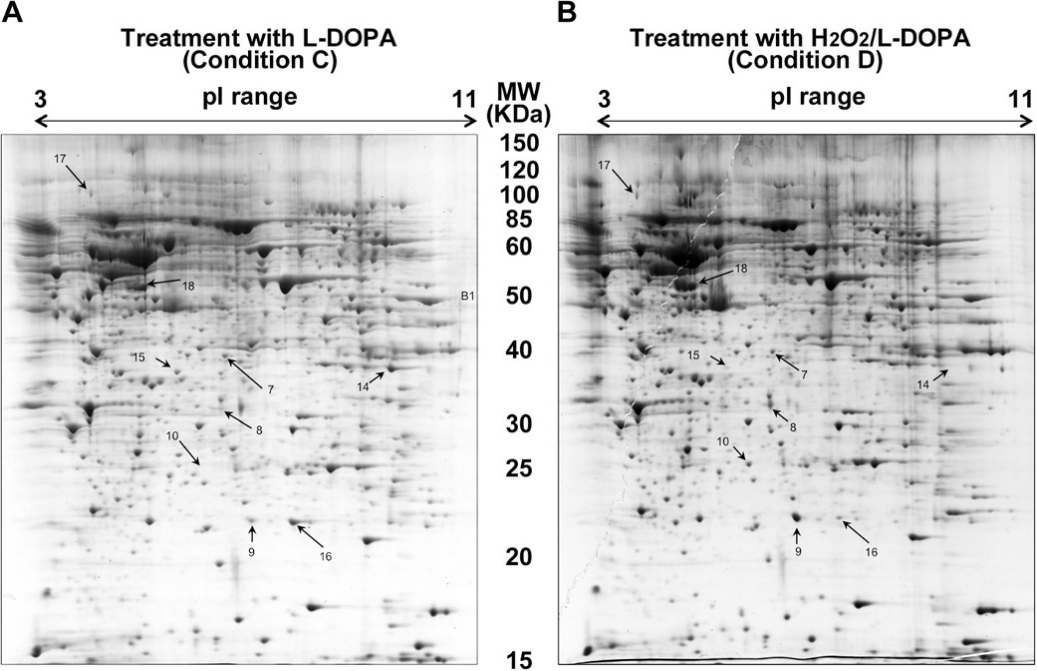
**Proteomes of SH-SY5Y cells exposed to L-DOPA with or without H**_**2**_ **O**_**2**_ **.** Representative 2-DE gels (n = 3 for each treatment) of the proteomes of SHSY5Y cells grown in media containing 200 μM L-DOPA for 8 hours in the **(A)** absence or **(B)** presence of 2 mM H_2_O_2_. The uppercase letters are used for those spots overrepresented in each condition whereas lowercase letters are used for spots underrepresented. The spots differentially represented are numbered and correspond to the proteins listed in Table 2.

Based on these findings we then performed a set of proteomic analyses on SH-SY5Y cells exposed to the same treatments as described above. The first experiment focused on the effects of H_2_O_2_ on the neuronal proteome where we compared the proteome profile of SH-SY5Y cells under control condition (Table 1; Condition A, Figure 2A) to cells treated with H_2_O_2_ (Table 1; Condition B, Figure 2B). In this experiment we observed significant up-regulation of ten proteins (spots 1–10) and significant down-regulation of six proteins (spots 11–16) compared to control condition (Table 1). In a second experiment we compared the proteome profile of cells exposed to L-DOPA (Table 1; Condition C, Figure 3A) to control cells and found one detectable protein (spot 17) that changes in response to L-DOPA exposure (Table 2). Finally, in a third experiment we compared the proteome profile from cells exposed to both H_2_O_2_ and L-DOPA (Figure 3B; Condition D) to control cells (Figure 2A; Condition A) and observed up-regulation of five proteins (spots 7–10) and down-regulation of four proteins (spot 14–16 and 18). Interestingly in this comparison spot 17 was detected in response to co-treatment with both H_2_O_2_ and L-DOPA but not in the control (Figures 2 and 3). Although L-DOPA can auto-oxidize we did not include catalase in these experiments as our findings show that L-DOPA treatment can reverse the decreased cell viability in response to oxidative stress suggesting the presence of the active form of L-DOPA in our experimental conditions. Detailed information on the number of spots detected in each gel is summarized in Table 1.

**Table 1:** **Treatment conditions of the SH-SY5Y neuronal cells**

Condition	Treatment regime	Number of spots detected in three gels	Spots matched to replicates	Spots matched to control	Figure no.
**A**	Control	1252, 1326, 1277	1212	-	2A
**B**	2 mM H_2_O_2_ - 8 hours	1287, 1202, 1219	1184	1033	2B
**C**	200 μM L-DOPA - 8 hours	1314, 1280, 1334	1257	1191	3A
**D**	Co-treatment with 200 μM L-DOPA and 2 mM H_2_O_2_ - 8 hours	1285, 1274, 1239	1215	1082	3B

**Table 2:** **Detailed information of spots up-regulated or down-regulated in each treatment condition**

Spot ID	Protein ID	Description	Mass	PI	Score	Protein coverage	AVG fold change	p-value	q-value	GO (Biological Process)
**1**	P07954	Fumarate hydratase, mitochondrial	54714	8.85	408	27.2	8.27	2.10E-03	4.68E-04	GO:0009056 ~ catabolic process, GO:0042592 ~ homeostatic process
**2**	Q16836	Hydroxyacyl-coenzyme A dehydrogenase	34613	8.76	118	21.5	4.81	3.22E-04	1.38E-03	GO:0055114 ~ oxidation reduction
**3**	Q9Y281	Cofilin-2	18764	7.66	113	33.1	7.4	2.75E-05	1.75E-03	GO:0030042 ~ actin filament depolymerization
**4**	P60981	Destrin	18852	8.14	147	26.1	5.08	1.62E-04	1.75E-03	GO:0006928 ~ cell motion, GO:0043243 ~ positive regulation of protein complex disassembly
**5**	P23528	Cofilin-1	18776	8.22	192	50.6	4.1	3.15E-03	1.99E-03	GO:0006928 ~ cell motion, GO:0043243 ~ positive regulation of protein complex disassembly
**6**	O00746	Nucleoside diphosphate kinase	17469	7.77	1024	41.4	2.7	3.71E-03	2.34E-03	GO:0009116 ~ nucleoside metabolic process
**7**	Q9BYZ2	L-lactate dehydrogenase A-like 6B	36817	7.62	244	29.6	4.52	1.02E-02	4.48E-03	GO:0055114 ~ oxidation reduction, GO:0006096 ~ glycolysis
**8**	P04083	Annexin A1	38816	6.57	142	6.62	1.88	3.49E-03	4.48E-03	GO:00070301 ~ cellular response to hydrogen peroxide, GO:0006954 ~ inflammatory response
**9**	Q99497	Protein DJ-1	20064	6.33	501	21.2	8.58	4.11E-04	4.48E-03	GO:0006979 ~ response to oxidative stress, GO:0042592 ~ homeostatic process
**10**	P30041	Peroxiredoxin-6	25109	5.74	348	35.7	12.7	5.85E-04	5.26E-03	GO:0009056 ~ catabolic process, GO:0055114 ~ oxidation reduction
**11**	P08670	Vimentin	53754	5.06	1536	62.2	0.08	2.21E-03	5.26E-03	GO:0006928 ~ cell motion
**12**	P67936	Tropomyosin alpha-4 chain	28620	4.65	138	23.8	0.07	2.47E-02	5.26E-03	GO:0006928 ~ cell motion
**13**	P09493	Tropomyosin alpha-1 chain	32746	4.69	405	33.1	0.12	2.37E-03	6.41E-03	GO:0006979 ~ response to oxidative stress, GO:0006928 ~ cell motion, GO:0046907 ~ intracellular transport
**14**	O14556	Glyceraldehyde-3-phosphate dehydrogenase	35953	8.49	1165	48.6	0.22	9.05E-03	1.04E-02	GO:0055114 ~ oxidation reduction, GO:0006096 ~ glycolysis, GO:0006928 ~ cell motion
**15**	Q9UBR2	Cathepsin Z	34658	6.13	308	15.7	0.18	4.90E-03	1.04E-02	GO:0006508 ~ proteolysis
**16**	Q08752	Peptidyl-prolyl cis-trans isomerase D	20436	6.77	139	8.88	0.12	9.20E-03	1.08E-02	GO:0043065 ~ positive regulation of apoptotic process
**17**	Q9Y4L1	Hypoxia up-regulated protein 1	1E + 05	5.09	192	27	N/A	N/A	N/A	GO:0042221 ~ response to chemical stimulus, GO:0006950 ~ response to stress
**18**	P10809	60 kDa heat shock protein	61105	5.72	289	29.6	4.77	8.26E-04	2.47E-02	GO:0032943 ~ mononuclear cell proliferation

**Figure 4:**
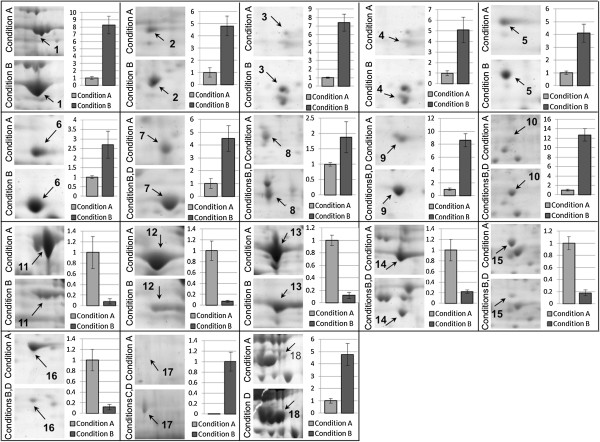
**Close-up view of protein spots differentially represented in response to H**_**2**_ **O**_**2**_ **, L-DOPA and H**_**2**_ **O**_**2**_ **/L-DOPA treatments.** Enlargements of gel portions containing the differentially abundant protein spots indicated in the gels shown in Figures 2 and 3. Column charts show the ratio for each differentially expressed protein. Differentially expressed proteins were studied using Student's t-test. The relative spot volume (Vol%) for proteins from the control condition was set to “1”.

We studied and classified the differentially expressed proteins according to their gene ontology (GO, with focus on biological process) through Uniprot (protein knowledge website: http://www.uniprot.org/) and QuickGO (http://www.ebi.ac.uk/QuickGO/). Several biological processes and pathways were affected due to the treatment conditions. For instance the majority of protein changes in response to oxidative stress belong to the “cell motion (GO:0006928)” category, most of which were reversed by L-DOPA co-treatment. More detailed information regarding the differentially expressed proteins are illustrated in Figure 4, summarized in Table 2 and discussed below.

### Oxidative stress changes metabolic routes

Glutathione is one of the main cellular antioxidants which prevents damage caused by ROS [17]. The reduced form of glutathione is oxidized during the conversion of reactive H_2_O_2_ into H_2_O by glutathione peroxidase and the oxidized form can be reduced back to its original state via NADPH and glutathione reductase. The main source of NADPH in cells is through the pentose phosphate pathway (PPP, also called the phosphogluconate pathway).

We observed a dramatic decrease in the expression of glyceraldehyde 3-phosphate dehydrogenase (GAPDH) in all conditions treated with H_2_O_2_ (spot 14 with an average of 0.22 fold change compared to the control) (Table 2). GAPDH catalyzes the conversion of glyceraldehyde 3-phosphate to D-glycerate 1,3-bisphosphate as part of glycolysis. GAPDH also acts as reversible metabolic switch under oxidative stress conditions where cells require excessive amounts of the antioxidant cofactor NADPH [18]. Indeed, it has been shown that oxidative stress causes an inactivation of GAPDH which re-routes the metabolic flux from glycolysis to the PPP [19]. This phenomenon in turn allows cells to produce higher levels of NADPH to overcome the oxidative conditions. Combined with our findings showing H_2_O_2_-mediated down-regulation of GAPDH, GAPDH modulation may have neuroprotective potential [20].

Unlike GAPDH, lactate dehydrogenase (LDH, spot 7, 4.52 fold change) is up-regulated in response to H_2_O_2_ exposure (Table 2). This cytosolic enzyme, involved in oxidation-reduction, can catalyze the conversion of pyruvate to lactate in the absence of oxygen [21]. However, LDH can also perform the reverse reaction in oxidative conditions and generate NADH.

Similar to the cytosolic routes, oxidative stress impacts the tricarboxylic acid (TCA) cycle as well as beta-oxidation of fatty acids in mitochondria. We detected substantial up-regulation of fumarate hydratase (spot 1, 8.27 fold increase) in response to H_2_O_2_, a member of the TCA cycle that catalyzes the reversible hydration/dehydration of fumarate to malate (Table 2). The TCA cycle generates NADH, which can either be used in respiration or be converted to NADPH via nicotinamide nucleotide transhydrogenase (NNT). Interestingly, the alteration of mitochondrial nucleoside-diphosphate kinase (spot 6, 2.7 fold increase), that catalyzes the exchange of phosphate groups between different nucleoside diphosphates, also supports the involvement of the TCA cycle in the oxidative stress response in neurons (Table 2).

Our analysis also showed up-regulation of 3-Hydroxyacyl CoA dehydrogenase (spot 2, 4.81 fold change), an oxidoreductase enzyme involved in beta-oxidation of fatty acids catalyzing oxidation of L-3-hydroxyacyl CoA by NAD + thus generating NADH in response to oxidative stress (Table 2).

### Neurons respond to oxidative conditions by enhancing cell survival mechanisms

In the oxidative stress conditions tested we observed up-regulation of proteins involved in cell survival such as Annexin A1, Peroxiredoxin-6 and PARK7/DJ-1 (Table 2).

Annexin A1 (spot 8, 1.88 fold) is known to be a calcium/phospholipid-binding protein that promotes membrane fusion [22]. This membrane-associated protein can regulate proliferation and apoptosis via the NF-κB signal transduction pathway [23, 24]. Annexin A1 can also directly improve cell survival by limiting excessive levels of ROS during oxidative stress in plant cells. It has been demonstrated that Annexin A1 from *Arabidopsis thaliana* (AnnAt1) is a redox sensor and displays peroxidase activity [25]. Moreover Rhee and co-workers have shown that, Annexin I serves as a stress protein in HeLa cells and that Annexins may constitute a new class of stress proteins [26].

Similarly, Peroxiredoxin-6, involved in redox regulation of the cell, is highly up-regulated (spot 10, 12.7 fold) in response to H_2_O_2_ exposure (Table 2). Peroxiredoxins represent antioxidants with the capacity to mediate signal transduction in mammalian cells [27]. Indeed, peroxiredoxin-6 can reduce H_2_O_2_ and short chain fatty acid, and phospholipid hydroperoxides [28]. Moreover, it has been reported that retinal ganglion cells over-expressing Peroxiredoxin-6 gain resistance against hypoxia-evoked generation of ROS and ROS-induced cellular insults by negatively regulating NF-κB-mediated death signaling [29].

The substantial up-regulation of PARK7/DJ-1 (spot 9, 8.58 fold), involved in protecting cells against oxidative stress and cell death, is not entirely surprising (Table 2). PARK7/DJ-1 has numerous reported functions, including anti-oxidative stress reactions, transcriptional regulation, and mitochondrial regulation [30]. Similar to Annexin A1 and Peroxiredoxin-6, PARK7/DJ-1 is also a stress sensor and its expression is increased upon oxidative stress and various other stresses [31, 32] playing a role in regulating the expression or stability of the mitochondrial uncoupling proteins SLC25A14 and SLC25A27 in dopaminergic neurons [33]. PARK7/DJ-1 is also a transcriptional co-activator that protects against neuronal apoptosis and it has been shown that its cytoprotective action is through inhibition of the p53-Bax-caspase pathway [34]. Moreover, it is known that PARK7/DJ-1 also enhances cell survival through the binding of Cezanne, a negative regulator of NF-kappaB [35].

It is also interesting to note that Cathepsin X (also known as Cathepsin Z, spot 15, ratio 0.18), involved in cell death, is attenuated (Table 2). This protein is a lysosomal cysteine proteinase and a member of the peptidase C1 family with both carboxy-monopeptidase and carboxy-dipeptidase activities [36]. It has been reported that α and γ enolases, showing neurotrophic activity, are molecular targets for Cathepsin X and that cleavage of C-terminal amino acids of α and γ enolases by Cathepsin X abolishes their neurotrophic activity affecting neuronal cell survival and neuritogenesis [37].

Down-regulation of Cyclophilin D (spot 16, ratio 0.12), the only human mitochondrial isoform of cyclophilins, is also notable (Table 2). Cyclophilin D is required for the formation of the mitochondrial permeability transition pore leading to cell necrosis [38]. In addition, opening of the mitochondrial permeability transition pore, orchestrated by Cyclophilin D, underlies oxidative stress-induced axonal degeneration [39]. Therefore attenuation of this protein in response to oxidative stress seems to play a pivotal role in cell survival.

### Cytoskeletal modifications by oxidative stress

Treatment with H_2_O_2_ resulted in modification of cell morphology in the absence of L-DOPA (Figure 1A). Indeed, the alteration of several structural proteins, in response to oxidative stress, indicates a rearrangement of the cytoskeleton. We observed up-regulation of all three members of the ADF/cofilin family including Cofilin-1 (spot 5, 4.1 fold), Cofilin-2 (spot 3, 7.4 fold) and Destrin, also known as ADF or actin depolymerizing factor (spot 4, 5.08 fold) (Table 2). Cofilins control actin polymerization/depolymerization in a pH-sensitive manner where they bind to actin filaments (F actin) and regulate actin cytoskeleton dynamics playing a critical role in the regulation of cytoskeletal organization and cell morphology [40].

Conversely, other actin binding proteins, such as Tropomyosin alpha-4 chain (spot 12, ratio 0.07) and Tropomyosin alpha-1 chain (spot 13, ratio 0.12) are down-regulated (Table 2). These proteins bind to actin filaments in muscle and non-muscle cells and play a central role in muscle contraction and in stabilizing cytoskeleton actin filaments in non-muscle cells [41].

The down-regulation of Vimentin (spot 11, ratio 0.08) under oxidative conditions is also remarkable (Table 2). Vimentin is attached to the endoplasmic reticulum, mitochondria and nucleus either laterally or terminally [42]. This cytoskeletal component plays an important role in anchoring organelles in the cytosol and is responsible for maintaining cell shape, cytoplasmic integrity, and stabilization of cytoskeletal interactions [43]. Cells deficient in Vimentin are therefore extremely delicate [43] suggesting that the observed change in neuronal cell morphology in response to H_2_O_2_ may be due to the observed attenuation of Vimentin (Table 2).

### L-DOPA promotes reversal of oxidative stress responses

**Figure 5:**
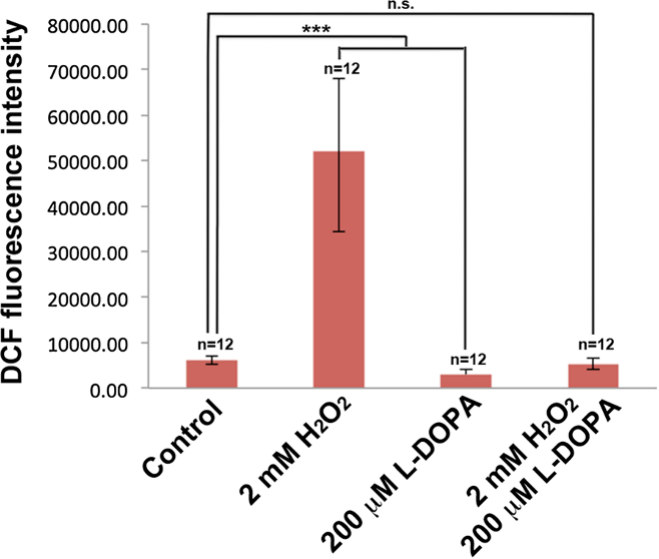
**Quantitative ROS measurements in response to H**_**2**_ **O**_**2**_ **, L-DOPA and H**_**2**_ **O**_**2**_ **/L-DOPA treatments.** Quantification of ROS production in SH-SY5Y cells in response to eight hours of 2 mM H_2_O_2_, 200 μM L-DOPA and 2 mM H_2_O_2_/200 μM L-DOPA treatments as determined by DCF fluorescence intensity. Each data point is the average of 12 replicate samples and presented as means ± SD. Standard deviations are indicated by error bars. ***p < 0.001.

An interesting phenomenon that was observed in our treatments is the ability of L-DOPA to reverse the effects of H_2_O_2_ on cellular morphology and cell viability (Figure 1). This reversal was also observed in terms of ROS where L-DOPA exposure reduced ROS levels, in the presence of H_2_O_2_, down to levels observed in control cells (Figure 5). Indeed, all the H_2_O_2_-induced protein alterations, affecting the cytoskeleton, were restored to baseline by supplementing the H_2_O_2_-containing media with L-DOPA (spots 3, 4, 5, 11, 12 and 13) (Figures 3B, Table 2). Furthermore, the induction of mitochondrial enzymes under oxidative stress condition (fumarate hydratase (spot 1), nucleoside-diphosphate kinase (spot 6) and 3-Hydroxyacyl CoA dehydrogenase (spot 2) was abolished in response to L-DOPA exposure (Table 2).

### Effects of L-DOPA

Only cells exposed to L-DOPA under oxidative stress conditions show significantly elevated expression of a 60-kDa heat shock protein (also known as HSP-60, spot 18, 4.77 fold overexpressed), a mitochondrial protein with cytoprotective functions [44]. This observation suggests that L-DOPA can mediate mitochondrial associated cell survival mechanisms as has been previously reported when exposing postnatal mesencephalic cultures, grown on glia monolayers, to L-DOPA in excess of 100 μM [45]. This heat shock protein contributes to the anti-apoptotic Hsp60/procaspase-3 complex and enhances cell survival [46]. It has been observed that mild Hsp60 deficiency primarily affects neuronal and/or glia cells, whereas more severe Hsp60 deficiency affects all tissues [47].

**Figure 6:**
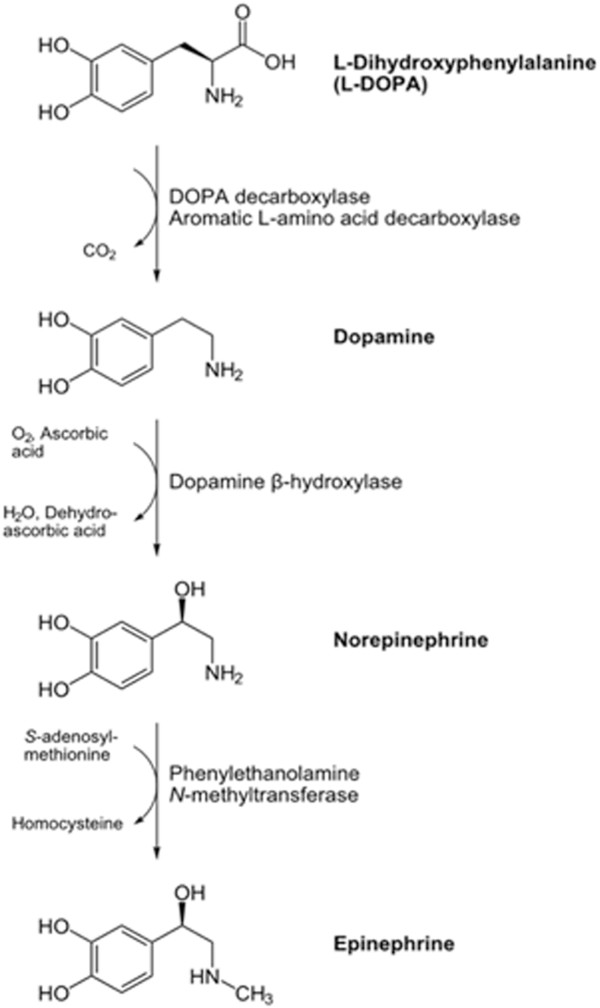
**Biosynthesis of catecholamines.** The conversion of L-DOPA to norepinephrine through dopamine requires molecular oxygen.

In addition to its effects on the cytoskeleton and mitochondria, L-DOPA seems to participate in a cell survival mechanism triggered by oxygen deprivation through induction of the hypoxia up-regulated protein 1 (ORP150, spot 17), a chaperone involved in protein folding [48]. We observed detectable levels of this protein only in the presence of L-DOPA regardless of the oxidative stress conditions (Table 2) suggesting that L-DOPA may aid hypoxia condition in cells. It has been shown in rat astrocytes that ORP150 is induced by hypoxia within 24 hours, augmented further during early re-oxygenation, and thereafter decreasing to baseline levels by 24 hours in normoxia [49]. Furthermore, the hypoxia-mediated induction of ORP150 appears specific as stress conditions such as heat shock, H_2_O_2_, cobalt chloride, 2-deoxyglucose, or tunicamycin does not affect ORP150 levels [49]. Interestingly, exposure to dopamine has similar ORP induction effects as hypoxia in PC12 cells [50]. In the catecholamine synthesis pathway dopamine is the first catecholamine synthesized from L-DOPA where norepinephrine and epinephrine are formed by further metabolic dopamine modifications (Figure 6). The conversion of dopamine to norepinephrine requires oxygen, thus the higher level of oxygen consumption due to L-DOPA exposure may cause cellular hypoxia and therefore induction of ORP150 with its concomitant cytoprotective effects.

Our findings, combined with previous data, suggest that although L-DOPA appears to influence neuronal cell survival pathways, this is most probably an indirect effect through the induction of endogenous antioxidant mechanisms [15].

## Conclusion

There is clear controversy surrounding L-DOPA effects associated with oxidative stress [16]. Using a 2DE-based proteomic study we have shown that oxidative stress changes neuronal metabolic routes and affects cytoskeletal integrity. Interestingly, L-DOPA reverses some of these H_2_O_2−_mediated effects at the cell morphology and cell viability level (Figure 1), at the ROS level (Figure 5) and at the protein level (Table 2). Exposure to L-DOPA may aid hypoxia condition in cells and therefore induction of ORP150 with its concomitant cytoprotective effects. However, L-DOPA exposure may simply evoke endogenous antioxidant mechanisms [15] and it cannot be ruled out that L-DOPA has direct ROS scavenging action [16]. Our study sheds light on the molecular interplay linking oxidative stress and L-DOPA in neuronal cells. Furthermore, the data presented here highlights the complex nature of L-DOPA in PD, demonstrating that additional studies are required that addresses L-DOPA action in the context of appropriate experimental design and choice of model mimicking PD.

## Methods

### Reagents and cell culture

Fetal bovine serum (FBS), phosphate buffered saline (PBS), Dulbecco’s minimum essential medium plus F12 (DMEM/F12), penicillin and streptomycin were purchased from Invitrogen (Gaithersburg, MD). Acrylamide/ bis-acrylamide, tris base, glycine, ammonium persulfate, PVDF membrane, TEMED, DTT, SDS, urea, thiourea, glycerol, ammonium bicarbonate, DMSO, ECL reagent, bromoplenol blue were purchased from Fisher Scientific (Pittsburgh, PA). Trypsin and trypan blue were obtained from Sigma-Aldrich (St. Louis, MO).

SH-SY5Y cells were cultured in DMEM/F12 containing 10% FBS, 100 U/ml penicillin and 100 mg/ml streptomycin at 37°C in an atmosphere containing 5% CO_2_. In order to perform treatments the media were supplemented with 2 mM H_2_O_2_ (Sigma-Aldrich, MO), 200 μM L-DOPA (Acros Organics, NJ), or a combination of H_2_O_2_/L-DOPA for eight hours (Table 1).

### Cell viability

Cell viability was evaluated by the neutral red uptake assay. 5×10^4^ SH-SY5Y cells/well were seeded in 96-well tissue culture plates and incubated overnight. The next day, the culture medium was removed and the cells were treated with either 2 mM H_2_O_2_, 200 μM L-DOPA and co-treated with 2 mM H_2_O_2_ and 200 μM L-DOPA in serum-free medium for eight hours. Untreated cells were used as control. Following incubation, cell morphology was recorded using an EVOS phase-contrast inverted microscope. The test-medium was then removed and cells were washed with PBS. Then 100 μl of a neutral red solution (40 μg/ml) was added in each well. The plates were incubated at 37°C for 2 hours. After incubation, the neutral red solution was removed and cells were washed with 150 μl PBS per well. Following this 150 μl neutral red destaining solution (50% ethanol, 49% deionized water, 1% glacial acetic acid) was added to the cells and the plates were rapidly shaken for at least 10 minutes. The absorbance was measured at 540 nm using an Epoch microplate spectrophotometer (BioTeck, VT). The results were reported as a percentage compared to control cells (considered as 100% viable).

### Detection of ROS formation

Intracellular ROS levels were detected by measuring the oxidation of the cell-permeable dye 2′, 7′-dichlorofluorescent diacetate (DCF-DA; Sigma-Aldrich, MO) to fluorescent DCF. To quantify intracellular ROS levels, 5×10^4^ SH-SY5Y cells/well were seeded in black 96-well plates. The next day, the culture medium was removed and the cells were incubated for 45 minutes with 200 μl of DCF-DA (final concentration 25 μmol)/well. After incubation cells were washed with PBS and exposed to 2 mM H_2_O_2_, 200 μM L-DOPA and 2 mM H_2_O_2_/200 μM L-DOPA in serum-free medium for eight hours as described above. The medium was then removed and cells were washed again with PBS. Relative fluorescence was measured using a GloMax®-Multi Detection System fluorescence plate reader (Promega, WI) at 485 nm excitation and 528 nm emission wavelengths.

### Protein sample preparation and 2-D gel electrophoresis

Triplicate batches of SH-SY5Y cells were seeded at 20–30% confluence and harvested when cell density reached 90%. After three washes with PBS, healthy cells were harvested from the plates and solubilized in 500 μl of lysis buffer: 8 M urea, 4% (w/v) 3-[(3-cholamidopropyl)dimethylammonio]-1-propanesulfonate (CHAPS), 0.5% (v/v) ampholytes (IPG buffer, GE Healthcare), 25 mM DTT and 0.002% bromophenol blue) and stored at −80°C. The insoluble fraction was discarded after centrifugation at 13,200 rpm for 5 min. The supernatant was collected and the protein concentration was determined according to the Bradford method, which showed a high reproducibility for this protein extraction protocol.

A solution containing 850 μg of soluble proteins in the sample buffer (same as lysis buffer but containing 2% CHAPS), was loaded onto 24-cm IPG strips (GE Healthcare), with non-linear (NL) pH 3–11 gradients. Focusing of proteins (using BioRad IEF instrument), equilibration of the focused IPG strips and the 12.5% SDS-PAGE for the second dimension (carried out in an Ettan Dalt Six apparatus (GE Healthcare)) were performed as previously described [51]. Briefly, proteins were focused at 20°C according to the following program: 1 h, 0 V and 12 h, 30 V (rehydration); 30-min gradient to 10,000 V; up to 9 h, 10,000 V until 85 kV-h. Focused IPG strips were equilibrated twice for 15 min in a buffer containing 50 mM Tris–HCl (pH 8.8), 6 M urea, 30% (v/v) glycerol, 2% (w/v) SDS, 0.002% bromphenol blue, and 1% (w/v) DTT. For the second equilibration step, DTT was replaced by 4.0% (w/v) iodoacetamide. In the second dimension 12.5% polyacrylamide SDS-PAGE gels were run in an Ettan Dalt Six apparatus (GE Healthcare) for 45 min at 3 watts/gel and then for 4 h at 18 watts/gel. Gels were stained with Colloidal Coomassie (CC) following the “blue silver” staining method (28), using 0.12% Coomassie Blue G-250 (Sigma), 10% ammonium sulfate, 10% phosphoric acid, and 20% methanol [52, 53].

### Analysis of differential protein abundance

Two-dimensional images were captured by scanning stained gels using an ImageScanner II (GE Healthcare) previously calibrated by using a grayscale marker (Eastman Kodak Co.), digitalized with Labscan 5.00 (v1.0.8) software (GE Healthcare), and analyzed with the ImageMasterTM 2D Platinum V7.0 software (GE Healthcare). Three gels for each condition, obtained from three independent cultures (biological replicates), were analysed to guarantee representative results. After automated spot detection, spots were checked manually to eliminate any possible artefacts, such as streaks or background noise. The patterns of each sample were overlapped and matched, using landmark features, to detect differentially expressed proteins. Variability in the number of protein spots detected among biological replicates was less than 10%, which may be due to experimental variability. Spot normalization (internal calibration to make the data independent from experimental variations between gels) was made using relative volumes to quantify the gel spots. Relative spot volume (Vol%) corresponds to the volume of each spot divided by the total volume of all the spots in the gel. Analysis of each differentially expressed protein between conditions was performed using Student's t-test and p < 0.05 was considered statistically significant [51, 54, 55]. False discovery rate (FDR) correction for multiple hypothesis testing was performed according to Storey’s method (q-value <0.05) [56]. Differentially expressed proteins were studied further when the ratio of the relative mean volume for one specific spot (in three biological replicates) was higher than 1.5 fold (the relative spot volume (Vol%) for proteins from the control condition was set to “1”).

### Protein identification by mass spectrometry

The protein spots of interest were manually excised from the gel and washed three times with ammonium bicarbonate/acetonitrile 1:1 (vol/vol) solution. Polyacrylamide fragments were dehydrated in acetonitrile and dried with a vacuum concentrator. Protein reduction and alkylation were performed by reswelling polyacrylamide fragments in 25 mM ammonium carbonate solution containing 0.15 mg/ml DTT at 56°C. This solution was replaced by a 25 mM ammonium bicarbonate solution containing 10 mg/ml 2-iodoacetamide (Bio-Rad) for 45 min at room temperature and in the dark. Fragments were dried as described above and tryptic cleavage was initiated by reswelling the gel in 25 mM ammonium bicarbonate solution containing 0.1 μg trypsin per gel slice (Promega, WI, USA) for 20 min on ice. The solution was then replaced by 25 mM ammonium carbonate solution and digestion carried out overnight at 37°C. Tryptic peptides were extracted at 37°C for 15 min with a 50% (vol/vol) acetonitrile and 10% (vol/vol) acetic acid solution. A second extraction was performed under the same conditions. A third extraction was performed at room temperature for 15 min with 100% acetonitrile. The pooled solution was dried under vacuum and resuspended in 10 μl 0.1% (vol/vol) formic acid solution.

Five microliters of each sample were loaded onto a 75 μm × 12 cm column self-packed with 3 μm ReproSil-Pur C18-AQ beads (Dr. Maisch), eluted with a gradient of 0–40% acetonitrile in 0.1% formic acid over 18 min at 300 nl/min using an Exigent nanoflow HPLC coupled directly to an LTQ-Orbitrap mass spectrometer (Thermo Fisher Scientific). Mass spectra were acquired in data-dependent analysis mode with one 60 000 resolution MS survey scan by the Orbitrap and up to eight concurrent MS/MS scans in the LTQ for the eight most intense peaks selected from each survey scan. Automatic gain control was set to 2 000 000 for Orbitrap survey scans and 5 000 for LTQ MS/MS scans. Survey scans were acquired in profile mode and MS/MS scans were acquired in centroid mode. Mascot generic format files were generated from the raw data using DTASuperCharge (version 1.01) for database searching. Mascot Generic Files combining MS and MS/MS spectra were automatically created for protein identification, and used to interrogate a nonredundant protein database using a local license of Mascot v 2.2 from Matrix Science through the Protein Global Server (GPS) v 3.6 (Applied Biosystems). The search parameters were set as follows: (i) NCBInr (2011.11.21) sequence database was used; (ii) taxonomy: All entries (16245521 sequences; 5585386883 residues); (iii) fixed and variable modifications were considered (Cys as S carbamidomethyl derivative and Met as oxidized methionine); (iv) one missed cleavage site was allowed; (v) precursor tolerance was 100 ppm and MS/MS fragment tolerance was 0.3 Da; (vi) peptide charge: 1+; and (vii) the algorithm was set to use trypsin as the enzyme. Additional criteria for confident identification were that the protein match should have at least 15% sequence coverage; for a lower coverage, only those proteins with a Mascot ions score above 54 and at least two peptides identified in the tandem MS analysis (with a significance level of p < 0.05), were considered valid.
